# Total hip and knee replacement surgery results in changes in leukocyte and endothelial markers

**DOI:** 10.1186/1476-9255-7-2

**Published:** 2010-01-19

**Authors:** Stephen F Hughes, Beverly D Hendricks, David R Edwards, Kirsty M Maclean, Salah S Bastawrous, Jim F Middleton

**Affiliations:** 1 Department of Biological Sciences, University of Chester, UK; 2 Haematology Department, North Wales (Central) NHS Trust, UK; 3 Haematology Department, North (West) Wales NHS Trust, UK; 4 Rheumatology Department, North Wales (Central) NHS Trust, UK; 5 Orthopaedics Department, North Wales (Central) NHS Trust. UK; 6 Leopold Muller Arthritis Research Centre, RJAH Orthopaedic Hospital, Medical School, Keele University, UK

## Abstract

**Background:**

It is estimated that over 8 million people in the United Kingdom suffer from osteoarthritis. These patients may require orthopaedic surgical intervention to help alleviate their clinical condition. Investigations presented here was to test the hypothesis that total hip replacement (THR) and total knee replacement (TKR) orthopaedic surgery result in changes to leukocyte and endothelial markers thus increasing inflammatory reactions postoperatively.

**Methods:**

During this 'pilot study', ten test subjects were all scheduled for THR or TKR elective surgery due to osteoarthritis. Leukocyte concentrations were measured using an automated full blood count analyser. Leukocyte CD11b (Mac-1) and CD62L cell surface expression, intracellular production of H_2_O_2 _and elastase were measured as markers of leukocyte function. Von Willebrand factor (vWF) and soluble intercellular adhesion molecule-1 (sICAM-1) were measured as markers of endothelial activation.

**Results:**

The results obtained during this study demonstrate that THR and TKR orthopaedic surgery result in similar changes of leukocyte and endothelial markers, suggestive of increased inflammatory reactions postoperatively. Specifically, THR and TKR surgery resulted in a leukocytosis, this being demonstrated by an increase in the total leukocyte concentration following surgery. Evidence of leukocyte activation was demonstrated by a decrease in CD62L expression and an increase in CD11b expression by neutrophils and monocytes respectively. An increase in the intracellular H_2_O_2 _production by neutrophils and monocytes and in the leukocyte elastase concentrations was also evident of leukocyte activation following orthopaedic surgery. With respect to endothelial activation, increases in vWF and sICAM-1 concentrations were demonstrated following surgery.

**Conclusion:**

In general it appeared that most of the leukocyte and endothelial markers measured during these studies peaked between days 1-3 postoperatively. It is proposed that by allowing orthopaedic surgeons access to alternative laboratory markers such as CD11b, H_2_O_2 _and elastase, CD62L, vWF and sICAM-1, an accurate assessment of the extent of inflammation due to surgery *per se *could be made. Ultimately, the leukocyte and endothelial markers assessed during this investigation may have a role in monitoring potential infectious complications that can occur during the postoperative period.

## Background

Involvement of the phagocytic leukocytes during an inflammatory response can be appreciated to be an important aspect of the innate (natural) immune response. During surgical procedures changes to the concentration of these circulating cell types (neutrophils and monocytes) can occur. A study by Wiik (2001) has demonstrated that abdominal surgery causes an increase in neutrophil and monocyte counts along with lymphocytopenia [1]. Høgevold *et al *(1999) have demonstrated that changes in leukocyte subpopulations occur in patients undergoing total hip replacement surgery. Specifically, the study involved twelve patients and found a leukocytosis, monocytosis, lymphocytopenia and granulocytosis after surgery [2]. Spark & Scott (2001) have also provided evidence to suggest that neutrophils play a critical early step in the development of the ischaemia-reperfusion syndrome, the systemic inflammatory response syndrome (SIRS) and sepsis following surgery [3].

With respect to orthopaedic surgery leukocyte and endothelial involvement as part of the post-operative period has not yet been extensively researched, particularly studies comparing a range of biological markers. Measurement of these parameters following lower limb orthopaedic surgery may therefore provide a useful tool as indicative markers following lower limb surgery.

The main aim of this pilot clinical study was to assess the effects of total hip replacement (THR) and total knee replacement (TKR) orthopaedic surgery on a range of leukocyte and endothelial markers. TKR involves using a tourniquet, creating a bloodless field for the surgeons to perform their work. During this time it can be appreciated that ischaemia-reperfusion injury may be incurred. Ischaemia is the reduction of blood supply to a part of the body and reperfusion occurs when blood flow is re-established. Ischaemia causes tissue injury, but it is during the period of reperfusion that extensive host tissue damage is proposed to occur, and has thus been termed ischaemia-reperfusion injury [4-10]. Ischaemia-reperfusion injury occurs in diseases such as ischemic heart disease, peripheral vascular disease and during surgical procedures, which involve the application of a tourniquet, such as upper limb (e.g. fasiectomy and carpal tunnel) and lower limb (e.g. knee arthroplasty and TKR) orthopaedic surgery [6,11-13]. It can be appreciated that during episodes of ischaemia-reperfusion injury an inflammatory response ensues, which would involve specific interactions between the phagocytic leukocytes and the vascular endothelium. This research investigation explored the role of leukocyte and endothelial markers in a clinical setting. THR and TKR surgery in general follow an uncomplicated course postoperatively, and it can be appreciated that the complication that surgeons fear most post-operatively are infections, as monitored by C-reactive protein (CRP) levels. However, little evidence is available to demonstrate the effects of orthopaedic surgery on other inflammatory markers, such as those of leukocytes and endothelial cells.

Therefore the study was undertaken to test the hypothesis that lower limb orthopaedic surgery results in changes to leukocyte and endothelial markers indicating inflammatory reactions postoperatively.

It is anticipated that any changes in the measured parameters may provide future direction with respect to therapeutic intervention. For example, if THR and TKR surgery results in prolonged leukocyte and endothelial activation, anti-adhesion molecules or free radical oxygen scavengers (e.g. anti-oxidants such as mannitol and vitamin E) may help reduce leukocyte and endothelial activation respectively, and thus reduce the inflammatory course postoperatively, which may have an important impact with regards to treatment strategies following orthopaedic trauma.

## Methods

### Subject Volunteers

Ethical approval for this study was permitted from the National Research Ethics Service (NRES). Ten volunteers scheduled for either elective THR or TKR surgery were recruited after informed consent. The test subjects were aged between 58 and 87 years old (mean age = 77 for both THR and TKR), and were all scheduled for elective surgery due to osteoarthritis. 5 patients were scheduled for THR (3 females and 2 males) and 5 patients for TKR (3 females and 2 males).

### THR surgery

Prior to surgery an 18GA cannula (BD VenflonTM, Sweden) was inserted into the arm at the ante-cubital fossa. A venous blood sample was then collected preoperatively, which stood as a baseline measurement for that particular patient. In theatre, patients were prepared for THR surgery by undergoing general anaesthesia. Blood samples were then collected from the arm by means of the cannula following surgery at day 1, 3 and 5 post-operatively. No tourniquet was used during this orthopaedic surgical procedure.

### TKR surgery

Prior to surgery an 18GA cannula (BD VenflonTM, Sweden) was inserted into the arm at the ante-cubital fossa. A venous blood sample was then collected preoperatively, which stood as a baseline measurement for that particular patient. In theatre, patients were prepared for TKR by undergoing general anaesthesia. Prior to commencing surgery the tourniquet was set around the upper thigh and inflated to 315 ± 9.80 mmHg, to ensure a bloodless field prior to surgery. The mean time of ischaemia was 94 ± 7.47 minutes per TKR surgical procedure. Blood samples were then collected from the arm by means of the cannula, upon the release of tourniquet at 5 and 15 minutes reperfusion, day 1, 3 and 5 post-operatively.

### Preparation of cell suspensions

Purified neutrophils and mononuclear cell suspensions were prepared by density gradient sedimentation on ficoll hypaque solutions as described by Lennie *et al*, (1987) [14]. Following isolation, cells were re-suspended in phosphate buffered saline (PBS) supplemented with di-potassium EDTA (1.5 mg/ml) to yield a final cell count of 2 × 10^6 ^cells/ml. All chemicals were supplied by Sigma-Aldrich, UK.

### Measurement of leukocyte concentration

Following venepuncture total leukocyte counts were performed using a Coulter^® ^MicroDiff[18] blood analyser (Beckman Coulter, UK).

### Measurement of cell surface expression of CD62L and CD11b

The monoclonal antibodies used were mouse anti-human CD62L (MCA1076F) and isotype-matched control IgG2b (MCA691F), mouse anti-human CD11b (MCA551F) and isotype-matched control IgG1 (MCA928F), and were purified immunoglobulin/fluorescein isothiocyanate (Ig/FITC) conjugates (AbD Serotec Ltd., U.K.). Following isolation of leukocyte subpopulations and adjustment of concentration (2 × 10^6 ^cells/ml), 10 μl of the monoclonal antibody (0.1 mg/ml) was added to 100 μl of the appropriate cell suspension. These were incubated at room temperature for 30 minutes, prior to assay analysis using flow cytometry of gated monocytes and neutrophils.

### Measurement of intracellular H_2_O_2 _production

Cells were isolated and intracellular H_2_O_2 _production was assessed by adaptation of a technique previously described by Bass *et al *(1983) [15]. The assay was based on the oxidation by H_2_O_2 _of non-fluorescent 2', 7'-dichlorofluoroscin diacetate (DCFH-DA) to stable and fluorescent dichlorofluorescein. H_2_O_2 _production was assessed in cells using a fixed volume of 0.5 ml cell suspension (2 × 10^6 ^cells/ml) mixed with 0.5 ml DCFH-DA (20 μM) in PBS. Cells were incubated in the dark, at 37°C for 30 minutes before immediate measurement using flow cytometry of gated monocytes and neutrophils.

### Measurement of plasma concentrations of leukocyte elastase

Blood samples were collected into EDTA tubes and were centrifuged at 1500 *g *for 10 minutes within 4 hours of blood collection. Plasma was removed and stored at -30°C. Quantification of human leukocyte elastase in subject plasma was carried out by ELISA using commercial kits provided by IBL (Hamburg, Germany) employing the method as initially described by Brower & Harpel (1983)[16].

### Measurement of plasma concentration of vWF and sICAM-1

Blood samples were collected into tri-sodium citrate tubes and were centrifuged at 1500 *g *for 10 minutes within 4 hours of blood collection. Plasma was removed and stored at -30°C. Quantification of vWF and sICAM-1 was subsequently measured by a two step enzyme immunoassay sandwich method. Measurement of the vWF parameter was performed using a Mini-Vidas automated immunoassay system that uses ELFA (Enzyme-Linked Fluorescent Assay) technology. The Mini-Vidas system and immunoassay kits were supplied from Biomerieux, UK. sICAM-1 was measured using commercial kits available from R&D Systems Europe (U.K).

### Statistical analysis

During this study, all results were presented as mean ± standard deviation (SD). Where data were normally distributed, repeated measures one-way analysis of variance (ANOVA) between samples test was employed adopting a 5% level of significance. Post hoc testing was conducted using the Tukey test for pairwise comparisons between means. Data that did not comply with normality were analysed using the Friedman test. Where the Friedman test resulted in statistical significance, subsequent tests were performed using the Wilcoxon test. Statistical significance was accepted when p ≤ 0.05.

Although no power calculations were performed, it is acknowledged that a limiting factor of this study was the relatively small number of patients recruited (n = 10). In order to fully appreciate the effects of surgery on the parameters measured more patients could have been recruited. This in-turn would have been beneficial to some of the statistical trends that were observed, that otherwise may have resulted in significant differences. It would also have been interesting to have followed up the patients with regards to measurement of their biological markers at review clinic's, this could have indicated any continued inflammatory reactions post surgery, which may have had an impact in supporting surgeons with their management strategies of patients during the post-operative period.

## Results

### Effect of THR and TKR surgery on leukocyte parameters

#### Leukocyte Count

**Figure 1:**
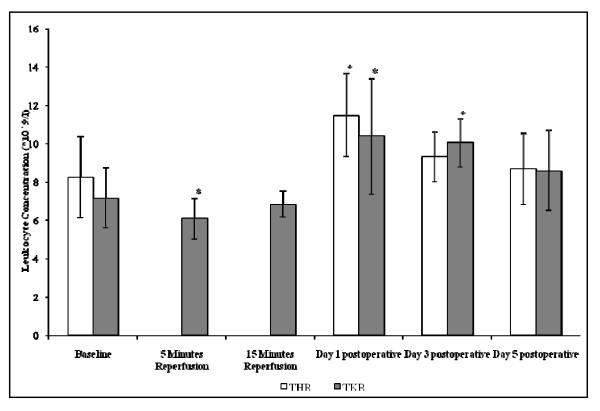
**Effect of THR and TKR surgery on total leukocyte concentration**. The points represent mean ± SD. p = <0.05 for THR and TKR, as determined by ANOVA and the Friedman tests respectively. p = 0.05 baseline vs day 1 postoperative THR, as determined by pairwise comparison testing; p = <0.05 baseline vs 5 minutes reperfusion, day 1 and day 3 postoperative TKR, as determined by the Wilcoxon test. (*, p < 0.05 compared to baseline).

Following THR and TKR surgery significant changes were seen in the total leukocyte concentrations (p = <0.05) (Figure 1). With regards to THR, the leukocyte concentration increased from baseline (8.24 ± 2.11) to day 1 postoperative (11.48 ± 2.17). The leukocyte concentration gradually decreased back towards basal levels at day 3 (9.30 ± 1.2) and day 5 (8.68 ± 1.86) postoperative. With respect to TKR surgery, the total leukocyte concentration decreased from baseline (7.16 ± 1.59) to 5 minutes reperfusion (6.08 ± 0.49). Total leukocytes then increased following 15 minutes reperfusion (6.84 ± 0.68) and peaked at day 1 postoperative (10.38 ± 3.01). By day 3 (10.04 ± 1.27) and day 5 (8.58 ± 2.15) postoperative the total leukocyte concentrations decreased toward basal levels.

### CD62L (L-selectin) expression

**Figure 2:**
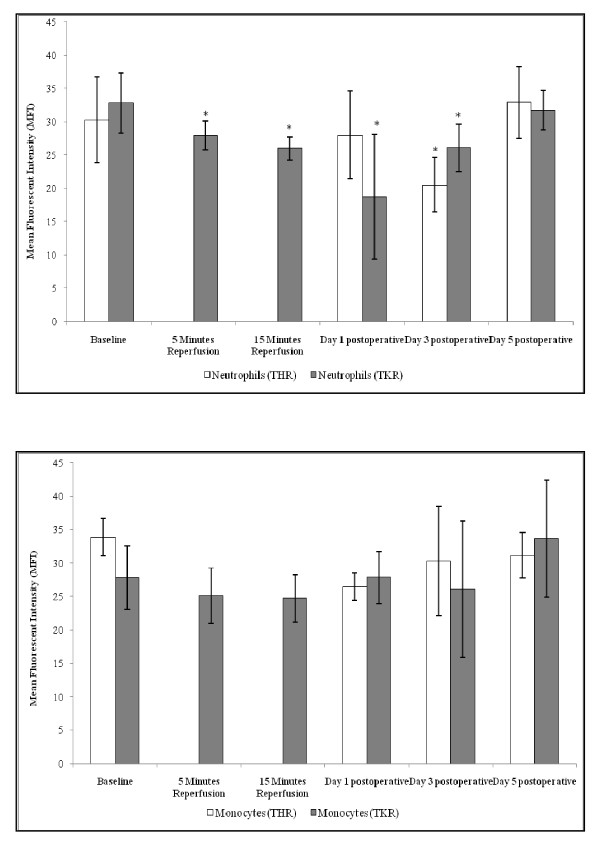
**Effect of THR and TKR surgery on CD62L cell surface expression of neutrophils (A) and monocytes (B)**. A, the points represent mean ± SD. p = <0.001 for neutrophils following THR and TKR surgery, as determined by ANOVA and the Friedman tests respectively. Baseline *vs *day 3 postoperative following THR p = 0.017, as determined by pairwise comparisons. p = <0.05 baseline *vs *5 and 15 minutes reperfusion, day 1 and day 3 postoperatively following TKR (Wilcoxon test). (* = p < 0.05 compared to baseline). B, the points represent mean ± SD. p = >0.05 for monocytes following THR and TKR surgery.

The results are expressed as mean fluorescent intensity (MFI) and represent the changes in the CD62L cell surface expression of neutrophils and monocytes following THR and TKR surgery (Figures 2a+b). Following THR surgery significant changes were seen in neutrophil CD62L cell surface expression (p = 0.003, as determined by ANOVA) (Figure 2a). This expression decreased from baseline (30.27 ± 6.42), during day 1 (28.01 ± 6.57) and day 3 (20.50 ± 4.06) postoperatively. CD62L cell surface expression increased above basal levels at day 5 postoperative (32.93 ± 5.35); pairwise comparison testing of these data showed significant differences between baseline *vs *day 3 postoperative (p = 0.017).

A significant decrease was seen in neutrophil CD62L cell surface expression following TKR surgery (p = 0.001, as determined by the Friedman test) (Figure 2a). This expression decreased from baseline (32.79 ± 4.49), during 5 minutes (27.93 ± 2.23) and 15 minutes (25.95 ± 1.76) reperfusion, with levels being at their lowest at day 1 postoperative (18.72 ± 9.39). CD62L expression on neutrophils gradually increased toward basal levels at day 3 (26.09 ± 3.58) and day 5 (31.71 ± 2.98) postoperatively. Upon further analysis the Wilcoxon test showed significant differences between baseline *vs *5 and 15 minutes reperfusion, day 1 and day 3 postoperatively (p = <0.05).

Although no significant changes were observed in the monocyte CD62L cell surface expression following THR surgery (p = 0.213, as determined by ANOVA) (Figure 2b), a trend of decreasing CD62L cell surface expression from baseline (33.86 ± 2.74) to day 1 postoperative was seen (26.45 ± 2.04). At day 3 (30.28 ± 8.17) and day 5 (31.12 ± 3.37) postoperative the CD62L cell surface expression on monocytes increased back toward basal levels.

Monocytes displayed a trend of decreasing CD62L cell surface expression from baseline (27.77 ± 4.75), during 5 (25.09 ± 4.11) and 15 (24.70 ± 3.51) minutes reperfusion following TKR surgery (Figure 2b). This expression increased towards or above basal levels at day 1 (27.81 ± 3.93), day 3 (26.03 ± 10.21) and day 5 (33.67 ± 8.76) postoperative, although no overall significant changes were observed (p = 0.281, as determined by ANOVA).

### CD11b expression

**Figure 3:**
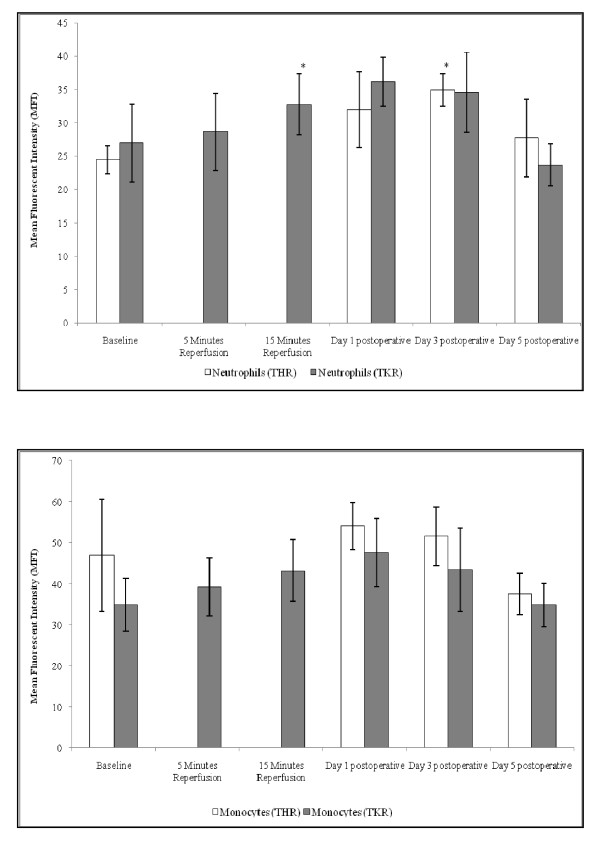
**Effect of THR and TKR surgery on CD11b cell surface expression of neutrophils (A) and monocytes (B)**. A, the points represent mean ± SD. p = <0.05 for neutrophils following THR and TKR surgery, as determined by ANOVA. Baseline *vs *day 3 postoperative following THR (p = 0.027, as determined by pairwise comparisons). Baseline *vs *15 minutes reperfusion, following TKR (p = 0.022, as determined by pairwise comparisons). (*, p < 0.05 compared to baseline). B, the points represent mean ± SD. p = 0.004 for monocytes following TKR, as determined by ANOVA.

Following THR surgery significant changes were seen in neutrophil CD11b cell surface expression (p = <0.05) (Figure 3a). Levels increased from baseline (24.49 ± 2.07), during day 1 (31.99 ± 5.67) and peaked at day 3 (34.95 ± 2.39) (p = 0.027) postoperatively, then decreased toward basal levels at day 5 postoperative (27.72 ± 5.82).

A significant increase was seen in neutrophil CD11b cell surface expression following TKR surgery (p = <0.05), (Figure 3a). This expression increased from baseline (27.00 ± 5.85), during 5 (28.66 ± 5.81) and 15 (32.80 ± 4.58) minutes reperfusion, peaking at day 1 postoperative (36.19 ± 3.68). CD11b expression on neutrophils gradually decreased toward basal levels at day 3 (34.61 ± 6.01) postoperatively, and was less than that of basal values at day 5 (23.70 ± 3.15) postoperative. Upon further analysis by pairwise comparison testing significant differences between baseline *vs *15 minutes reperfusion (p = 0.022) was observed.

Although no significant changes were observed in the monocyte CD11b cell surface expression following THR surgery, a trend of increasing CD11b expression from baseline (46.90 ± 13.72) to day 1 postoperative was seen (54.01 ± 5.81). At day 3 (51.55 ± 7.2) postoperative the CD11b cell surface expression on monocytes decreased toward basal levels, and at day 5 (37.5 ± 5.09) postoperatively the CD11b expression was lower than that of basal levels (Figure 3b).

Monocytes displayed a significant increase in CD11b cell surface expression (p = 0.004) from baseline (34.82 ± 6.45), during 5 (39.20 ± 7.05) minutes, 15 (43.11 ± 7.54) minutes reperfusion, and peaking at day 1 postoperatively (47.62 ± 8.31) following TKR surgery. CD11b expression decreased toward basal levels at day 3 (43.36 ± 10.21) and day 5 (34.85 ± 5.33) postoperative (Figure 3b).

The CD11b cell surface expression on monocytes was consistently higher than that seen in neutrophils following both THR and TKR surgery, which may be due to the fact that monocytes are larger that neutrophils and express more CD11b on their surfaces.

### Intracellular H_2_O_2 _production

**Figure 4:**
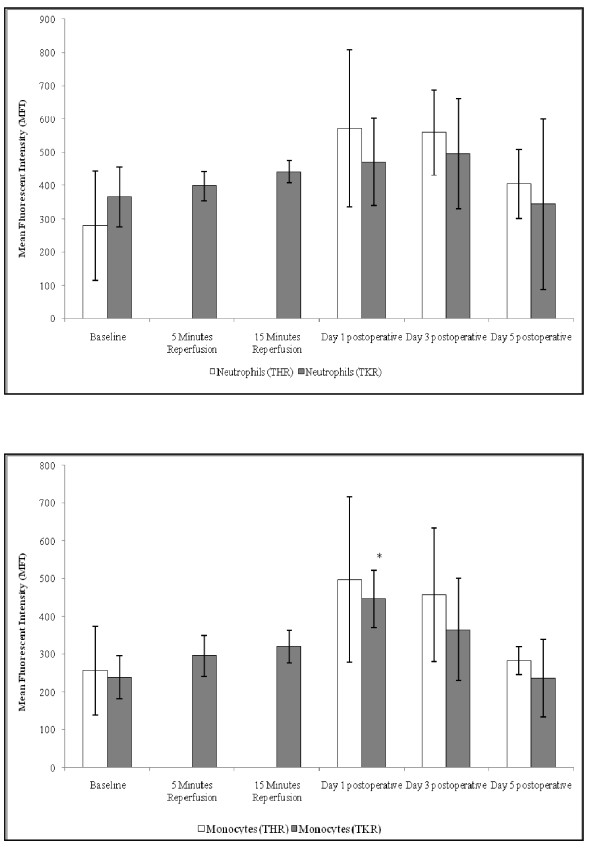
**Effect of THR and TKR surgery on intracellular H_2_O_2 _production of neutrophils (A) and monocytes (B)**. A, the points represent mean ± SD. p = 0.035, as determined by ANOVA following THR surgery. B, the points represent mean ± SD. p = 0.002, as determined by ANOVA following TKR surgery. Baseline *vs *day 1 postoperative following TKR (p = 0.011, as determined by pairwise comparisons) (*, p < 0.05 compared to baseline).

Following THR surgery significant changes were seen in neutrophil intracellular H_2_O_2 _production (p = 0.035) (Figure 4a). Levels increased from baseline (281 ± 164) peaking at day 1 (572 ± 236) postoperatively. Intracellular H_2_O_2 _production decreased toward basal levels at day 3 (559 ± 128) and day 5 postoperative (405 ± 104).

A trend of increasing neutrophil intracellular H_2_O_2 _production from baseline (365 ± 90), during 5 minutes (398 ± 44), 15 minutes (441 ± 34) reperfusion, day 1 (471 ± 131) and peaking at day 3 (496 ± 165) postoperatively was observed following TKR surgery (Figure 4a). The intracellular H_2_O_2 _production in neutrophils decreased below basal levels at day 5 postoperatively (344 ± 255). These differences in neutrophil intracellular H_2_O_2 _production following TKR surgery were not significant.

Although no significant changes were observed in the monocyte intracellular H_2_O_2 _production following THR surgery (Figure 4b), a trend of increasing intracellular H_2_O_2 _production from baseline (257 ± 118) to day 1 postoperative was seen (497 ± 219). At day 3 (457 ± 177) and day 5 (283 ± 34) postoperative H_2_O_2 _levels in monocytes decreased toward basal levels.

Monocytes displayed a significant increase in intracellular H_2_O_2 _production (p = 0.002) from baseline (239 ± 56), during 5 (296 ± 55) and 15 (320 ± 44) minutes reperfusion, and peaking at day 1 postoperatively (446 ± 75) (p = 0.011) following TKR surgery (Figure 4b). The intracellular H_2_O_2 _production of monocytes then decreased toward basal levels at day 3 (365 ± 135) and day 5 (236 ± 103) postoperatively.

#### Leukocyte elastase

**Figure 5:**
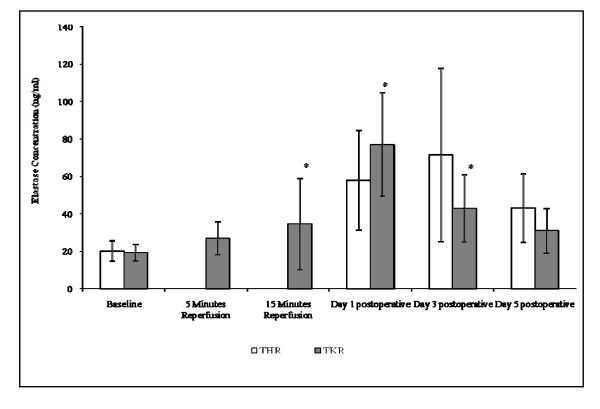
**Effect of THR and TKR surgery on elastase concentration**. The points represent mean ± SD, p = 0.003 for TKR surgery, as determined by the Friedman test. p = <0.05 baseline *vs *15 minutes reperfusion, day 1 and day 3 postoperative, as determined by the Wilcoxon test. (*, p < 0.05 compared to baseline).

Although no significant changes were observed in the elastase concentration following THR surgery (Figure 5), a trend of increasing elastase concentration from baseline (20.16 ± 5.46), during day 1 postoperative (57.94 ± 26.73), peaking at day 3 postoperative (71.52 ± 46.34) was seen. At day 5 (43.16 ± 18.19) postoperative the elastase concentration following THR surgery decreased toward basal levels. Following TKR surgery significant changes were seen leukocyte elastase concentrations (p = 0.003) (Figure 5). Leukocyte elastase concentrations increased from baseline (19.20 ± 4.52), during 5 (26.81 ± 9.01) and 15 (34.44 ± 24.52) (p < 0.05) minutes reperfusion, and peaked at day 1 (77.00 ± 27.80) (p < 0.05) postoperatively. It decreased toward basal levels at day 3 (42.98 ± 18.05) and day 5 (30.88 ± 12.08) postoperatively, although still remained at a higher level to those of basal values (p < 0.05 for day 3).

#### Effect of THR and TKR orthopaedic surgery on endothelial markers

##### vWF

**Figure 6:**
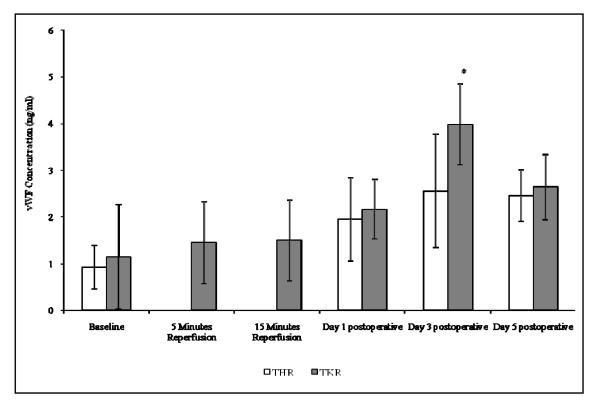
**Effect of THR and TKR surgery on vWF concentration**. The points represent mean ± SD, p = <0.001 TKR surgery, as determined by ANOVA. Baseline vs day 3 postoperative for TKR surgery (p = <0.05), as determined by pairwise comparison tests. (*, p < 0.05 compared to baseline).

The results are expressed as ng/ml and represent the changes in vWF concentration following THR and TKR surgery (Figure 6). This parameter was measured as a marker of endothelial activation. Although no significant changes were observed in the vWF concentration following THR surgery (p = 0.08, as determined by ANOVA), a trend of increasing vWF concentration from baseline (0.93 ± 0.46), during day 1 (1.95 ± 0.89) and peaking at day 3 postoperative was seen (2.56 ± 1.22). At day 5 (2.46 ± 0.55) postoperative the vWF concentration decreased marginally and remained two fold higher to that of basal values.

With regards to TKR surgery (Figure 6) significant changes were observed in vWF concentrations (p = <0.001, as determined by ANOVA). vWF concentrations increased from baseline (1.15 ± 1.12), during 5 (1.45 ± 0.88) and 15 (1.50 ± 0.87) minutes reperfusion, at day 1 (2.16 ± 0.64), and peaking at day 3 (3.98 ± 0.86) postoperatively. vWF concentration decreased at day 5 (2.64 ± 0.70) postoperatively, although remained at a higher level to those of basal values (2 fold). Upon further analysis pairwise comparison testing showed significant differences between baseline *vs *day 3 postoperatively (p < 0.05).

##### sICAM-1

**Figure 7:**
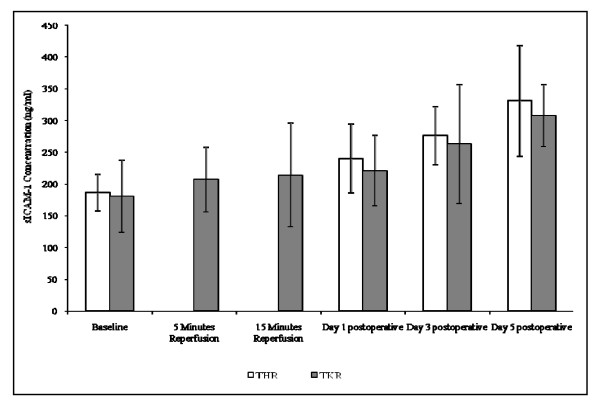
**Effect of THR and TKR surgery on sICAM-1 concentration**. The points represent mean ± SD, p = 0.032 for THR surgery, as determined by ANOVA.

The results are expressed as ng/ml and represent the changes in sICAM-1 concentration following THR and TKR surgery (Figure 7). This parameter was measured as marker of endothelial activation. Following THR surgery significant changes were seen in sICAM-1 concentrations (p = 0.032, as determined by ANOVA). sICAM-1 concentration increased from baseline (186.90 ± 29.12), during day 1 (240.17 ± 54.67), day 3 (275.71 ± 46.24), and peaked at day 5 (330.72 ± 87.44) postoperatively. Although no significant changes were observed in the sICAM-1 concentration following TKR surgery (p = 0.068, as determined by the Friedman test), a trend of increasing sICAM-1 concentration from baseline (180.28 ± 57.45), 5 (207.11 ± 51.25) and 15 (214.00 ± 82.88) minutes reperfusion, day 1 (221.20 ± 55.70), day 3 (263.94 ± 94.78) and day 5 (307.85 ± 49.52) postoperative was seen (Figure 7).

## Discussion

Results from the study demonstrated evidence of increased leukocytosis following THR and TKR surgery. Specifically, THR surgery resulted in increased total leukocyte counts, peaking at day 1 postoperatively, and although this appeared to be decreasing at day 5 postoperatively it still remained higher to those of basal values (pre-operative). Similar patterns were observed following TKR surgery. The results obtained during this study complement previous studies which provided evidence of leukocytosis following various surgeries such as total hip replacement surgery, and provide further evidence of increased leukocytosis up to day 5 post THR and TKR surgery [2,3]. It may therefore be appreciated that following long-bone surgical intervention there is a systemic response resulting in leukocytosis. These changes possibly take effect due to increased bone marrow turnover which has resulted from THR and TKR surgery procedures, postoperative wound and tissue repair, or probably due to a combination of these contributing factors.

During this clinical study there was a significant effect on neutrophil CD62L expression following both THR and TKR surgery. Similar trends were also observed in monocytes following both THR and TKR surgery, although these did not reach statistical significance. CD62L cell surface expression decreased from baseline (preoperatively), up to day 3 (THR) and up to day 1 (TKR). This was in agreement with Fassbender *et al*, (1999) who also reported a decrease in leukocyte CD62L expression following THR [17]. Interpretation of the results from the present study suggests that there was increased shedding of CD62L from the cell surface of neutrophils following THR and TKR surgery. This evidence indicates that CD62L may play a role during the early rolling stages of the leukocyte adhesion cascade and provides further evidence that monocytes follow a similar pattern post-surgery, which may facilitate leukocyte adhesion to the vascular endothelium during the acute inflammatory response following surgery.

Another element of the current investigations was to ascertain whether THR and TKR surgery resulted in changes in the cell surface expression of the CD11b adhesion molecule. There was a significant effect of THR and TKR surgery on the CD11b cell surface expression of neutrophils and monocytes (TKR surgery only). Results demonstrated an increase in CD11b expression from baseline (preoperative) up to day 3 postoperatively (THR) and up to day 1 (TKR) for both neutrophils and monocytes. This expression in monocytes was consistently higher than that seen in neutrophils. The up-regulation of CD11b was evident in both the phagocytic leukocytes (neutrophils and monocytes), and suggests that CD11b on these cells may be binding to counter-receptors, such as ICAM-1 present on the surface of vascular endothelium. This would occur as part of the inflammatory response post-orthopaedic surgery, where increased ICAM-1 may be due to elevated production due to endothelial activation. In agreement with others who demonstrated an increased neutrophil CD11b expression following upper limb surgery [6], this present study complements their findings and provides further evidence of monocytic involvement (represented by increased CD11b expression) during the acute phase response following both THR and TKR surgery.

Increased leukocyte adhesion to the vascular endothelium during an inflammatory response is associated with cell activation [18,19]. During the present study leukocyte activation following THR and TKR was assessed by measuring the intracellular production of H_2_O_2 _by neutrophils and monocytes. Both these cells displayed a significant increase in the intracellular production of H_2_O_2_, from baseline (preoperatively) up to day 1 postoperatively for neutrophils and monocytes following THR and TKR respectively. These findings are in accord with CD11b results which also suggested that neutrophils and monocytes were activated over a similar time period. Neutrophils displayed increased intracellular production of H_2_O_2 _compared to monocytes, suggesting that neutrophils may be more efficient in performing the respiratory burst to that of monocytes during an acute phase response post surgery.

In addition to changes to H_2_O_2 _production, during leukocyte activation it can be appreciated that further bioactive material, such as superoxide and elastase are released extracellularly [20,21]. Therefore to support the evidence of increased leukocyte activation following THR and TKR surgery measurement of leukocyte elastase was performed. A significant increase in the leukocyte elastase levels were displayed from baseline (preoperatively) up to day 1 post-surgery following TKR, with levels decreasing at day 3 and 5 postoperatively. Evidence of increased leukocyte elastase has also been reported using a human model of tourniquet-induced forearm ischaemia-reperfusion injury, where elastase levels increased from baseline, during 10 minutes ischaemia and up to 15 minutes reperfusion [5].

Collectively, the actions of the degradative substances H_2_O_2 _and elastase may potentially cause damage to host tissue following major orthopaedic surgery. Measurements of the intracellular production of H_2_O_2 _and elastase by phagocytic leukocytes may therefore provide a useful marker that could be applied to monitoring post-operative complications and clinical outcome after TKR or THR.

Endothelial activation following THR and TKR surgery was assessed via measurement of vWF and sICAM-1 concentrations, which are established markers of endothelial activation [22-24]. During the present study, significant changes in vWF concentration following TKR surgery were evident, with an increase from baseline up to day 3 postoperative and similarly for THR surgery although not significant. A significant increase in sICAM-1 was also demonstrated from baseline (preoperative) up to day 5 postoperative following THR surgery, with a similar trend being observed following TKR surgery. Data obtained from this study suggest that there is an increased liberation of vWF from the storage organelles of the vascular endothelium following surgery, and that ICAM-1 may be up-regulated and is being shed into the blood. The up-regulation of ICAM-1 fits with the increased levels of CD11b expression by leukocytes, which may facilitate leukocyte-endothelial cell interactions following orthopaedic surgery. In comparison to a study performed by Klimiuk *et al *(2002), who demonstrated increased serum concentrations of sICAM-1 and sE-selectin in patients with rheumatoid arthritis, the current study provides further evidence of increased sICAM-1 levels following major orthopaedic surgery [24]. Fedi *et al*, (1999) measured vWF levels before and during THR and TKR surgeries yet found no significant changes, however their study did not investigate postoperatively the effects of surgery on vWF levels [25]. The present study provides further evidence that significant changes to vWF levels do occur following TKR, especially after 3 days, and suggests that this parameter may provide useful marker for monitoring endothelial activation following joint replacement surgery.

During THR, no tourniquet is used and the operated area is always well vascularised, and clamping and diathermia is only used to stop surgical bleeding. TKR surgical procedures involve the application of a tourniquet, which allows a bloodless field for the surgeons to perform their work. Despite this difference, both tourniquet (TKR) and non-tourniquet (THR) applied orthopaedic surgery produced similar changes in leukocyte and endothelial markers.

It is proposed that changes in the measured biological parameters during this study may not be due to a single factor, but due to a number of factors that may result following surgery such as: tissue damage, wound repair, introduction of foreign material (e.g. prosthesis) and ischaemia-reperfusion injury during THR replacement surgery.

Changes in the leukocyte and endothelial markers are indicative of increased inflammatory reactions after orthopaedic trauma. It is therefore proposed that leukocyte and endothelial markers such as CD62L, CD11b, H_2_O_2, _elastase, vWF and sICAM-1, may provide an alternative method for monitoring an acute inflammatory response postoperatively, in comparison to the conventional method of measuring CRP. Infections, including those that are subclinical, are a complication after orthopaedic surgery and CRP has limitations in its use. Other markers that could monitor or predict infections are lacking. Therefore the leukocyte and endothelial markers described in the present study could be useful in this regard.

## Conclusion

It appears that changes in the leukocyte and endothelial markers following THR and TKR surgery followed a similar pattern. In general most of the markers measured during these studies peaked between days 1-3 postoperatively, when the most noticeable changes occurred. Changes in CD62L, CD11b, H_2_O_2_, elastase, vWF and sICAM-1 levels may therefore have clinical implications for understanding the development of inflammatory responses post surgery.

## Competing interests

The authors declare that they have no competing interests.

## Authors' contributions

SFH carried out the isolation of leukocyte sub-populations, assessment of leukocyte adhesion, assessment of total leukocyte counts, assessment of leukocyte activation and the immunoassays. BDH performed assessment of the inflammatory response. SSB participated in the design and collection of the blood sampling procedure. DRE and KMM advised on the clinical implications. SFH and JFM supervised the study, participated in its design and coordination and drafted the manuscript. All authors read and approved the final manuscript.
